# Negative frequency dependent selection on plasmid carriage and low fitness costs maintain extended spectrum β-lactamases in *Escherichia coli*

**DOI:** 10.1038/s41598-019-53575-7

**Published:** 2019-11-20

**Authors:** Tatiana Dimitriu, Frances Medaney, Elli Amanatidou, Jessica Forsyth, Richard J. Ellis, Ben Raymond

**Affiliations:** 1 University of Exeter, Penryn campus, Penryn, Cornwall, TR10 9FE UK; 2 School of Biological Science, Royal Holloway University of London, Egham, Surrey TW20 0EX UK; 3 Surveillance and Laboratory Services Department, Animal and Plant Health Agency, APHA Weybridge, Addlestone, Surrey KT15 3NB UK

**Keywords:** Evolutionary genetics, Antimicrobial resistance, Bacterial genes

## Abstract

Plasmids may maintain antibiotic resistance genes in bacterial populations through conjugation, in the absence of direct selection pressure. However, the costs and benefits of conjugation for plasmid and bacterial fitness are not well understood. Using invasion and competition experiments with plasmid mutants we explicitly tested how conjugation contributes to the maintenance of a plasmid bearing a single extended-spectrum ß-lactamase (ESBL) gene (*bla*_*CTX-M-14*_). Surprisingly, conjugation had little impact on overall frequencies, although it imposed a substantial fitness cost. Instead, stability resulted from the plasmid conferring fitness benefits when rare. Frequency dependent fitness did not require a functional *bla*_*CTX-M-14*_ gene, and was independent of culture media. Fitness benefits when rare are associated with the core plasmid backbone but are able to drive up frequencies of antibiotic resistance because fitness burden of the *bla*_*CTX-M-14*_ gene is very low. Negative frequency dependent fitness can contribute to maintaining a stable frequency of resistance genes in the absence of selection pressure from antimicrobials. In addition, persistent, low cost resistance has broad implications for antimicrobial stewardship.

## Introduction

Understanding the selection pressures that maintain antimicrobial resistance (AMR) genes is key to understanding how resistance will respond to antibiotic usage and how we might develop interventions to extend the use of antibiotics in the face of resistance. Importantly, AMR gene frequencies show a varied and unpredictable response to reduced rates of usage: in some cases, resistance declines readily after antibiotics are withdrawn, in other cases resistance genes persist effectively in the absence of recent selection^1–3^. Moreover, even when selection pressure is intense AMR genes may not rise to fixation^4^. Understanding the fate of antibiotic resistance genes when antibiotic use is reduced or withdrawn is key to developing effective resistance management or ‘antibiotic stewardship’ strategies, especially those that rely on cycling of antimicrobials^5^.

The situation is further complicated by the role of bacterial plasmids, which are responsible for a substantial proportion of clinically important resistance and for the accumulation of multi-drug resistance by many of the most difficult to treat nosocomial bacteria^6^. Plasmids can maintain themselves, and the AMR genes they carry, as genetic parasites in the absence of direct selection pressure from antibiotics^7^. If rates of horizontal transfer (conjugation) are sufficient to offset fitness cost of carriage or loss from segregation, then plasmids may be stable or capable of increasing in frequency in bacterial populations without selection from antibiotic usage^2,8^.

In this study, we sought to explicitly test the importance of conjugation for stable maintenance of the IncK plasmid pCT in *Escherichia coli* in the absence of antibiotic selection. This plasmid carries a single resistance gene - the extended-spectrum ß-lactamase (ESBL) *bla*_*CTX-M-14*_ - which confers resistance to penicillins and third generation cephalosporins^9^. Invasion and competition experiments used wild type (highly conjugative) and conjugation-null plasmid mutants and a blue/white marker system based on inactivation of ß-galactosidase to track plasmid transfer (see Methods) and cost of engaging in conjugation. In addition we tested the contribution of a functioning *bla*_*CTX-M-14*_ for the fitness, persistence and stability of pCT and non-conjugative vectors (see Table 1 for a full list of mutants).

**Table 1:** Bacteria and plasmids used in this study.

Designation	Characteristics	Reference/source
K12 MG1655	Wild type *E. coli*	*E. coli* Genetic Stock Center #7740
MG1655 Δ*lacZYA*	Δ*lac* mutant ‘White’ on Xgal & IPTG	Medaney *et al*.^12^
pCT	Highly conjugative wild type plasmid	Cottell *et al*.^10^
pCTΔ*trbA*	Conjugation null mutant	this study
pCT*bla*_CTX-M-14_::*aph*	kanamycin resistant mutant with disrupted ESBL resistance gene	Cottell *et al*.^10^
pCTΔ*trbA bla*_CTX-M-14_::*aph*	non-conjugative plasmid in which ESBL gene is replaced with kanamycin resistance	this study
pZS*2 R	non-conjugative vector carrying kanamycin resistance	Chuang *et al*.^36^
pZS- *bla*_CTX-M-14_	vector engineered to express ESBL resistance	this study

## Results and Discussion

**Figure 1:**
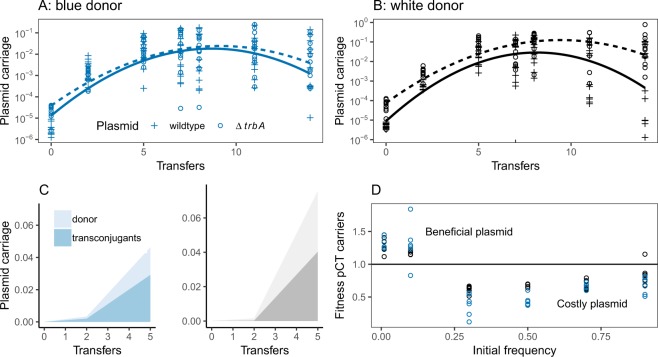
The invasion dynamics of wild type pCT plasmids (+symbols and solid lines) and conjugation null mutants (Δ*trbA* mutant – open circles and dashed lines) in transfer experiments in complex nutrient rich LB broth initiated with a low frequency of plasmid carriers. Experiments were repeated using initial plasmid donors in the chromosomal MG1655 wild background (**A** – ‘blue donor’ with blue data and fitted models) and in the MG1655 Δ*lac* mutant background (**B** – ‘white donor with black data and fitted models). Fitted models are quadratic glms with transfer and transfer^2^ as covariates. **C** The relative contribution of transconjugants (darker shading) and plasmids carriers having the original donor background (lighter shading) to the total bacterial population. Data are mean proportions from the 12 wild type plasmid replicates only, the blue panel shows data from blue donor and the grey panel data from white donors. **D** Relative fitness of MG1655 bacteria carrying the Δ*lac* mutant over different initial frequencies, fitness >1 indicates beneficial plasmid carriage. Experiments were repeated with blue and white donors, as above.

We expected that conjugation would enable plasmid persistence in the absence of antibiotics, or even facilitate the invasion of plasmids from low to high frequencies. However, experimental data showed that the pCT plasmid consistently increased in frequency regardless of ability to conjugate (Fig. 1A,B). Conjugation did increase plasmid frequencies overall (mixed model -likelihood ratio = 8.16, *df* = 1, *P* = 0.0043), but this effect was small when compared to the increase in frequency over several orders of magnitude through time (likelihood ratio = 322, *df* = 1, *P* < 0.0001). Plasmid frequencies declined after reaching a maximum of 10^−2^ −10^−1^ (Fig. 1A,B) (significant quadratic term- time^2^ likelihood ratios = 37.9 *df* = 1, *P* < 0.0001). In the wild type pCT transconjugants made up a substantial proportion of plasmid carriers after only five transfers (Fig. 1C), indicating that the similarities between conjugative and non-conjugative plasmids were not the result of low conjugation. The genetic background of the initial plasmid donor (wildtype or Δ*lacZYA*) had no impact on plasmid dynamics (Likelihood ratio = 1.97, *df* = 1, *P* = 0.16).

The pCT plasmid is highly persistent, and previously, the mechanism accounting for persistence was inferred to be a near-zero fitness cost of carriage^10^. However, the dynamics of invasion from rare seen here, and the subsequent decline from high frequencies, are better explained by pCT conferring a negative frequency dependent (NFD) fitness advantage to its host, where fitness is higher when a genotype is present at lower frequencies in the population. Competition experiments using our conjugation null pCT∆*trbA* mutant confirmed that carriage of this plasmid was beneficial at low frequencies and costly at high frequencies (Fig. 1D; *F*_*1,70*_ = 35.0, *P* < 0.001). As above, there was no effect of bacterial background on relative fitness (*F*_*1,69*_ = 1.6, *P* = 0.21). Moreover, these fitness experiments indicate that equilibrium carriage (when plasmid free and plasmid carrying bacteria have equal fitness) is in the region of 10^−1^ – 10^−2^ cells, which corresponds to the maximum frequencies in the transfer experiment (Fig. 1A,B). In the early transfers we are able to show the relative contribution of conjugation and fitness effects to total carriage of wild type pCT (Fig. 1C). Importantly, even without conjugation, the original plasmid carriers were able to increase in frequency from 1 in 10^5^ to 1 in 50 cells.

**Figure 2:**
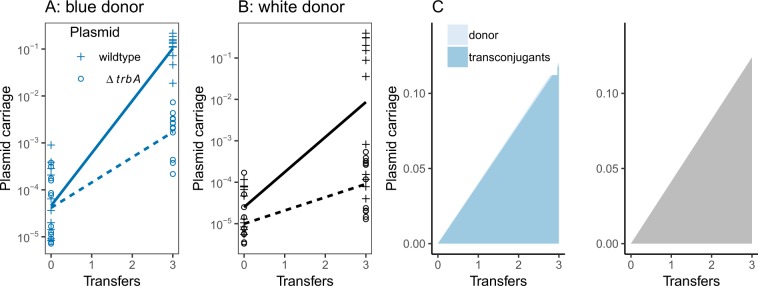
The invasion dynamics of wild type pCT plasmids (+symbols and solid lines) and conjugation null mutants (Δ*trbA* mutant – open circles and dashed lines) in minimal media initiated with a low frequency of plasmid carriers in the (**A**) blue donor background and (**B**) white donor background (**C**) The relative contribution of transconjugants (darker shading) to the total bacterial population. Plasmids carriers having the original donor background (lighter shading) have also been plotted but these values are too low to be visible (max proportion = 0.002). Data are mean proportions from the 12 wild type plasmid replicates only. As in Fig. 1 the blue panel shows data from blue donor and the grey panel data from white donors.

The focal pCT plasmid has few non-essential genes that are not required for transfer and maintenance: the typical IncI backbone accounts for most gene content^9^. Nevertheless, poorly characterized genes could encode metabolic functions responsible for frequency dependency fitness via resource competition^11^. However, plasmid carrying cells could also invade from rare in minimal media (M9 broth), where complex resources are absent (Fig. 2A,B). In addition, very high numbers of transconjugants appeared after only three transfers, suggesting a higher level of conjugation rate in minimal media (Fig. 2C). We measured pCT conjugation in both media in a separate experiment using a marked recipient strain, and confirmed that conjugation frequencies are approximately 30-fold higher in M9 than in LB (Fig. S1). Consistent with this observation, in minimal media the wild type plasmid increased in frequency more rapidly than the non-conjugative mutant (Fig. 2; time*plasmid interaction; Likelihood ratio = 32.2, *df* = 1, *P* < 0.0001), while there was no such effect in complex media (Fig. 1; time*plasmid interaction; Likelihood ratio = 2.54, *df* = 1, *P* = 0.11).

**Figure 3:**
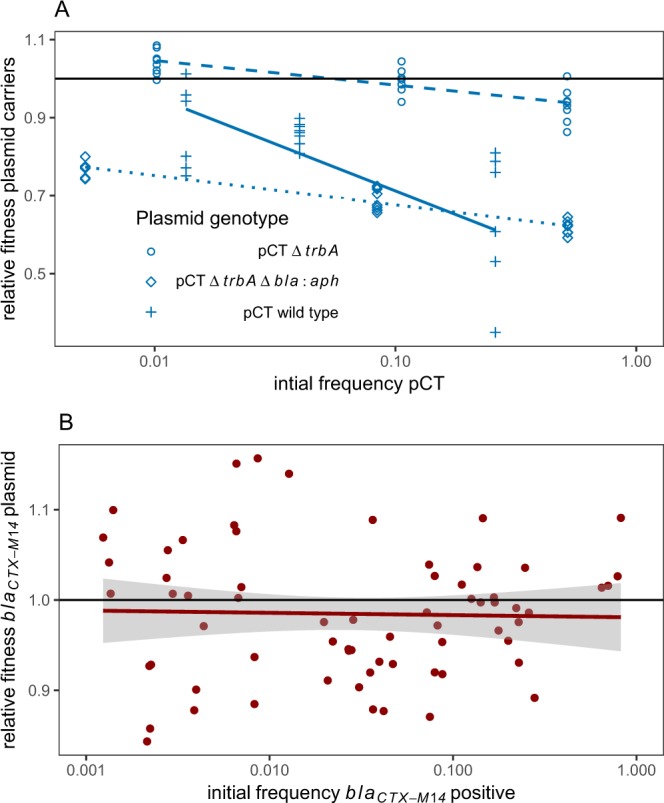
(**A**) Frequency-dependent fitness of WT plasmids in comparison to conjugation null pCTΔ*trbA* and double mutants also lacking the *bla*_*CTX-M-14*_ beta-lactamase gene pCTΔ*trbA*Δ*bla*::*aph*. All three genotypes show strong negative frequency dependent fitness (*F*_*1,208*_ = 19.9, *P* < 0.0001) and there is a strong effect of genotype on average relative fitness (*F*_*2,206*_ = *86.5, P* < 0.0001). Fitness data were generated independently to experiments in Fig. 1. (**B**) Impact of *bla*_*CTX-M-14*_ and initial frequency on fitness costs of carriage of non-conjugative vector pZS*2 *R* in competition experiments. Data points are independent repeats with a fitted linear model ± SE. Frequency did not affect the relative fitness of plasmids with and without this ESBL gene (*F*_*1,65*_ = *1.48, P* = *0*.23). The relative fitness of resistant plasmids carrying *bla*_*CTX-M-14*_ was estimated at 0.985 (confidence interval 0.967–1.002).

Next, we investigated the importance of the resistance gene *bla*_*CTX-M−14*_ on bacterial fitness and patterns of NFD. Replacing the *bla*_*CTX-M-14*_ with a kanamycin resistance cassette substantially increased the fitness burden associated with pCT∆*trbA* (Fig. 3A, reduction in fitness relative to pCT∆*trbA* -0.18, *t* = *−*10.7, *P* < 0.0001), indicating that the fitness cost of *bla*_*CTX-M-14*_ might be particularly low. Knock-in genetic manipulation with a cloning vector showed that the fitness cost of *bla*_*CTX-M-14*_ was indistinguishable from zero in conventional competition experiments (Fig. 3B). Detoxifying ß-lactamase resistance, including that conferred by *bla*_*CTX-M-14*_ can confer NFD fitness to resistant bacteria in the *presence* of ß-lactam antibiotics^12–14^. Conceivably, this gene might provide an NFD advantage in the absence of antibiotic if it were detoxifying another substrate. However, deleting *bla*_*CTX-M-14*_ did not abolish the NFD fitness of conjugational null pCT∆*trbA* (Fig. 3A). Knock-in experiments also confirmed that NFD fitness was not associated with *bla*_*CTX-M-14*_ (Fig. 3B).

Evidence that conjugation determines a substantial proportion of the fitness costs imposed by plasmids has been indirect^15–17^. Here we used the wild type pCT and pCT∆*trbA* to assess the fitness cost associated with conjugation directly. This cost was substantial (Fig. 3, reduction in fitness relative to pCT∆*trbA* -0.15, *t* = −6.57, *P* < 0.0001). These competition experiments can explain why the loss of ability to conjugate had a minimal impact on plasmid dynamics: the gain in competitive fitness accompanying loss of conjugation compensates very closely for loss of ability to move between bacterial lineages. Only in minimal media was there a gain in plasmid level fitness for maintaining conjugation (Fig. 2). Experiments with diverse plasmids indicate that conjugation can readily offset the costs of plasmid carriage^7^, in this study the benefits of conjugation could only just offset the costs of conjugation in nutrient rich media. A high cost of conjugation can explain the existence of natural conjugation-null mutations in plasmids encoding virulence factors in *Bacillus anthracis* or multi-drug resistance in *E. coli*^18,19^.

Importantly, NFD was not sufficient to allow plasmid invasion under all circumstances. This mechanism can only increase frequency for plasmids encoding low cost resistance genes. For example, the mutant carrying kanamycin resistance could not have invaded populations as carriers always had a relative fitness less than that of plasmid-free cells (Fig. 3). NFD fitness has widespread implications for the maintenance of low cost AMR genes outside the laboratory, and could stabilize gene frequencies of resistance when drugs are withdrawn. The latest surveillance data in England shows that resistance to third generation cephalosporins in *E. coli* bacteraemia remained stable at 10%, despite considerable efforts to reduce prescription rates for these drugs^20^, while frequencies of particular resistant lineages such as ST131 also show strong stability^21^. Another potential outcome is that selection *against* plasmids at high frequencies might stabilize AMR gene frequencies, at least in the face of modest selection pressure. Important caveats here are that continuous selection from antibiotic usage can favour the transfer of genes to the chromosome, and this has been documented for *E. coli*^22–24^. In addition, data on prevalence of ESBL genes in Asia suggests that the frequency of these genes can reach very high levels (50–70%) under strong selection^25^.

Many plasmid encoded traits such as ß-lactamases, Cry toxins and detoxifying mercury resistance confer NFD fitness via social interactions in structured environments^14,26,27^, while colicin plasmids can confer positive frequency dependent fitness^28^, as well as negative density dependent fitness^29^. A wide range of secreted bacterial virulence factors are encoded on mobile elements, and these are commonly inferred to be cooperative public goods^30–32^. Current theory suggests that social traits accumulate on plasmids because horizontal transfer provides a means of reducing competition from cheaters^32,33^ or a means of increasing genetic relatedness^30^. An alternative, non-mutually exclusive explanation is that plasmids accumulate genes that drive frequency dependence, and that social interactions are just one route to this phenomenon. The existence of both positive and negative frequency dependent selection on plasmids suggests that fluctuating selection pressure is the important common factor here. Recent genomic analyses suggest that NFD may be widespread for accessory genes in *Streptococcus pneumonia* and *E. coli* and that mobile elements contain the majority of genes at intermediate frequency likely to drive NFD^21,34^. Mechanisms could include bacteria-host, bacteria-bacteria or bacteria-MGE interactions in addition to niche specialization. Here we show that bacteria-bacteria interactions are sufficient to produce strong NFD.

### Methods

The strains used in this study are *E. coli* K-12 MG1655 and a MG1655 Δ*lacZYA* mutant. The MG1655 *lac* operon deletion was described previously^12^ and produced a *Δlac* mutant with a white phenotype in the presence of X-Gal & IPTG as opposed to the blue wild type phenotype. The focal plasmid in this study is pCT, an IncK plasmid, which carries the ESBL resistance gene *bla*_CTX-M-14_, which confers effective resistance to the third generation cephalosporin antibiotic cefotaxime^9^. We produced a conjugation null mutant of pCT via disruption of *trbA*, which encodes a putative regulation protein, at position 38848–40110 on pCT, as it is indispensable for plasmid transfer^35^. This mutant was produced by the Xercise protocol^36^. Diagnostic PCR was used to assess the insertion and subsequent deletion of the dif-CAT-dif fragment into MG1655. Diagnostic PCR was conducted using Taq (Qiagen, Manchester, UK) with an annealing temperature of 55 °C and using the primer F TrbA diag (5′-CGGCATCCAGGCAGGCATCA-3′) and R TrbA diag (5′-TTCAGCCCTGCCCGGTCATT-3′). Deletion used the primers F-TrbA del (5′–TTCTGCATCAACGGTATCAACAAGCACCGTTTCAGTTATTTCAGTGT GCTGGAATTCGCCCT-3′) and R-TrbA del (5′-ATTGTTCGCATTAATTCCACTC AGCCTCATCCCGAAATTTATCTATTTATCTGCAGAATTCGCCCTTCCT-3′). Electrocompetent cells were prepared according to the Bio-Rad protocol, and transformed with both pCT and pCT*ΔtrbA* plasmids using MicroPulser electroporation apparatus (Bio-Rad). Phenotypic confirmation of loss of conjugative ability was confirmed using mating experiments^37^ and no trans-conjugants were identified from transfer experiments using pCT*ΔtrbA* in this study. Additional plasmid mutants include a CTX-M-14 deletion mutant, disrupted by insertion of a kanamycin resistant cassette^10^, a gift of Laura Piddock (Univ. of Birmingham). To assess the impact of the *bla*_*CTX-M-14*_ gene on fitness, this gene was cloned into the pZS*2 R vector^38^, a low copy plasmid with a pSC101* replication origin and a kanamycin resistance marker. Amplification of *bla*_*CTX-M-14*_ from pCT used primers containing a XhoI restriction site and this product was ligated with pZS*2 R plasmid after digestion with XhoI. pZS-*bla* plasmids were transformed into MG1655; pZS*2 R control plasmid was transformed into MG1655 *ΔlacZYA*.

Plasmids were transformed into both host backgrounds. There is no detectable fitness difference between the wild type and the *lacZYA* knockout in broth (mean relative fitness of wild type = 1.006, SE = 0.02, *n* = 32); this fitness is also unaffected by relative frequency (F_1,31_ = *0.0001, P* = 0.99). The plasmid transfer experiment replication used six replicates per treatment using wild type pCT and the conjugation null mutant (Δ*trbA*). Transfers were initiated with two initial proportions of carriers, approximately 0.0001–0.00001, although these treatments had no impact on plasmid dynamics and were pooled in the analyses here. Transfer experiments were duplicated by switching the plasmid carrier strain between the wildtype and the Δ*lacZYA* ‘white’ mutant and competing plasmids with the opposing blue or white background genotype. The transfer regime used 1000x dilution into 1 ml of Lysogeny Broth (LB) in 24-well plates- transfers took place every 2–3 days. In transfer and competition experiments the proportion of blue and white mutants, as well as the frequency of pCT in both backgrounds was monitored by dilution plating cultures onto LB agar containing X-gal and IPTG (with and without ampicillin 100 μg/ml) each week. A replicate experiment used transfer in M9 broth and ran for 3 transfers only. To evaluate conjugation frequencies during the transfer experiment, we mixed the Δ*lacZYA* pCT donor strain and a MG1655 spontaneous mutant resistant to rifampicin^39^ in 50/50 ratios and measured donor, recipient and transconjugant densities after 24 h on ampicillin (100 μg/ml), rifampicin (100 μg/ml) and ampicillin + rifampicin respectively.

### Measurement of bacterial fitness

Fitness measurements were calculated in competition experiments. Initially, separate cultures were prepared in overnight cultures of 1 ml LB broth (37 °C, 180 rpm). Competing cultures were then mixed at desired ratios and diluted 1:100 into fresh broth and co-cultured for 24 h to homogenize the physiology of competitors. Densities and relative frequencies (day1) were calculated by dilution plating and mixtures were again diluted at 1:100 and cultured for 24 hours. Measurement of frequencies on day 2 and day 1 were used to calculate relative growth rate (log_e_(final proportion/(initial proportion*100), relative fitness is then calculated as the ratio of the relative growth rates of the two competing strains^40^. By convention fitness was expressed as the relative growth of plasmid carriers/plasmid null strains, or of antibiotic resistant/antibiotic susceptible competitors.

### Data analysis

The serial transfer experiments were designed such that a linear mixed-effects (lme) model could be used to analyze the resulting data, with the proportion of plasmid carriers as the response variable; time, plasmid type, donor type and transfer regime as fixed effects; and replicate as a random effect. Statistical modelling was conducted in *R* v3.2.2 using the *nlme* package^41^. Restricted maximum likelihood (REML) lme models were converted to maximum likelihood (ML) models to allow model comparison using mixed model ANOVA via Likelihood ratio tests or the Aikaike Information Criterion (AIC). Fitness experiments were analysed using generalized linear modeling, planned post hoc comparisons used treatment contrasts.

## Supplementary information

Figure S1

## Data Availability

The research data supporting this publication are openly available from the University of Exeter's institutional repository at: 10.24378/exe.2043. Mutants are available on request from BR.
