# Supplementary material for: Negative frequency dependent selection on plasmid carriage and low fitness costs maintain extended spectrum β-lactamases in Escherichia coli
Source: Sci Rep. 2019 Nov 20;9:17211. doi: 10.1038/s41598-019-53575-7 (PMC6868128; doi:10.1038/s41598-019-53575-7)

Supplementary Information for “Negative frequency dependent selection on plasmid carriage and low fitness costs maintain extended spectrum β-lactamases in *Escherichia coli.”*

Tatiana Dimitriu, Frances Medaney, Elli Amanatidou, Jessica Forsyth, Richard J. Ellis, Ben Raymond.

Figure S1: Comparison of pCT conjugation frequency in LB and M9 media. MG1655 Δ*lacZYA* pCT and MG1655 Rif^R were mixed in a 50/50 ratio and diluted 1000-fold in conditions similar to the transfer experiment (Figure 1). Strains frequencies were measured by selective plating after 24h. A shows the proportion of transconjugants in the total population and B shows the frequency of conjugation measured as the density of transconjugants per donors (T/D) after 24h.

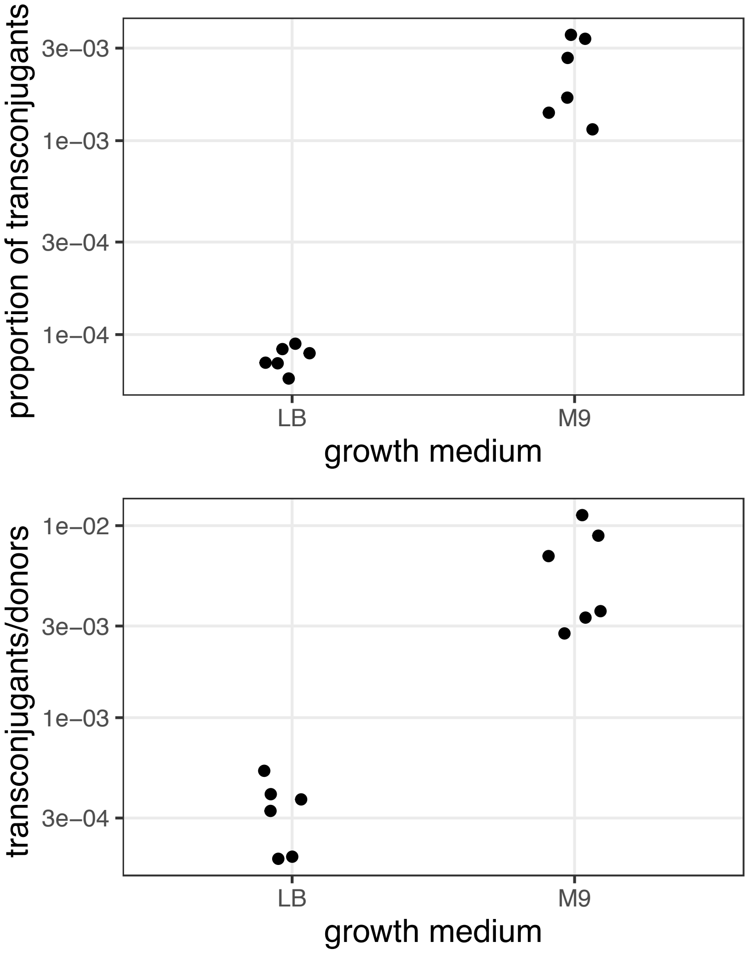

Supplement: Supplementary file 1 — Figure S1 [file 41598_2019_53575_MOESM1_ESM.pdf]
